# Supplementary material for: Phytochemical Analysis of Acacia ehrenbergiana (Hayne) Grown in Qatar: Identification of Active Ingredients and Their Biological Activities
Source: Molecules. 2022 Sep 28;27(19):6400. doi: 10.3390/molecules27196400 (PMC9571875; doi:10.3390/molecules27196400)

Supplementary File

Phytochemical Analysis of *Acacia ehrenbergiana* (Hayne) Grown in Qatar: Identification of Active Ingredients and Their Biological Activities

Vandana Thotathil, Hanan Rizk, Ameena Fakhroo and Lakshmaiah Sreerama*

Phytochemical analysis of the Isolated Compounds

The acetone fractions were subjected to HPTLC and HPLC analysis. Out of nine fractions, three pure compounds were isolated and subjected to IR and NMR analysis for further identification.

COMPOUND 1 (AE-HX-001-P1)

**SPECIFICATON**	**SPECTRUM**
Probable structure	Stigmasterol 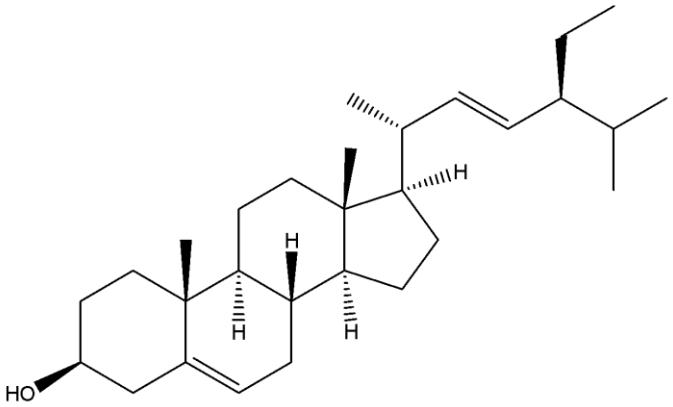
CAMAG HPTLC Scanner III<br>Mobile Phase: Hex: EtoAc (7:3)<br>Rf: 0.86<br>λ max: 206nm	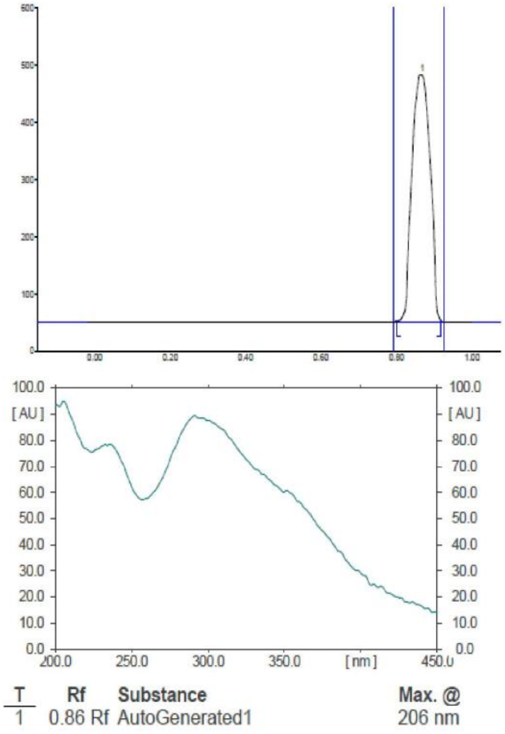

UV spectrum<br>PDA detector: 200-800nm<br>λ max: 193nm	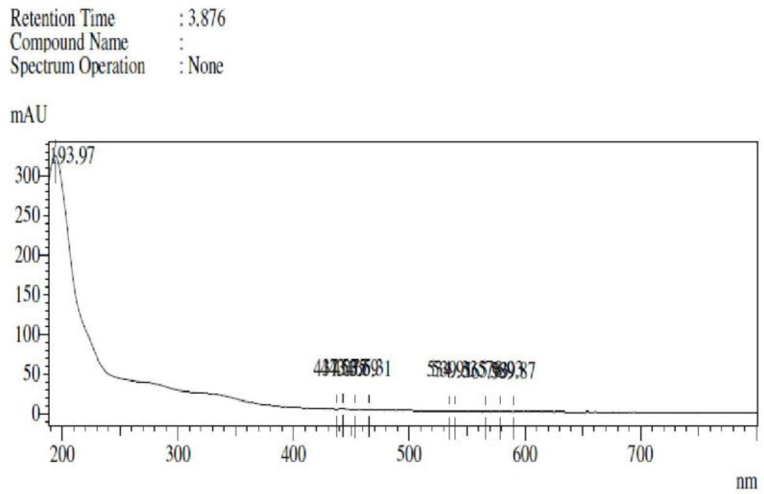
SHIMADZU SPD-20A HPLC<br>Column: Enable C_{18}, 4.6x250mm (5micron)<br>Mobile Phase: ACN:H_2O (15:85) with 0.1% H_3PO_4<br>λ max: 280nm<br>Inj. Vol: 20µl<br>Flow rate: 1.0ml/min<br>Rt: 3.876min	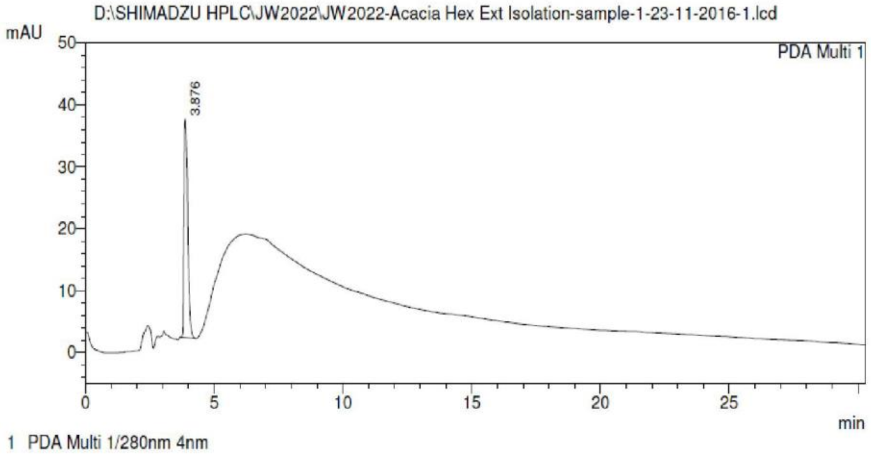
Bruker FT-IR<br>Wavelength scan: 500-4000cm^{-1}	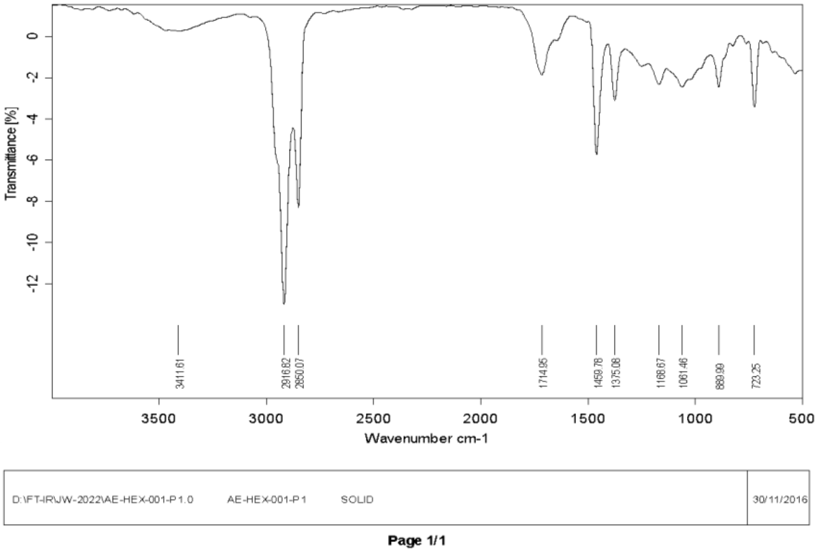

BRUKER NMR 400MHz
^{1}HNMR($CDCl_3$):

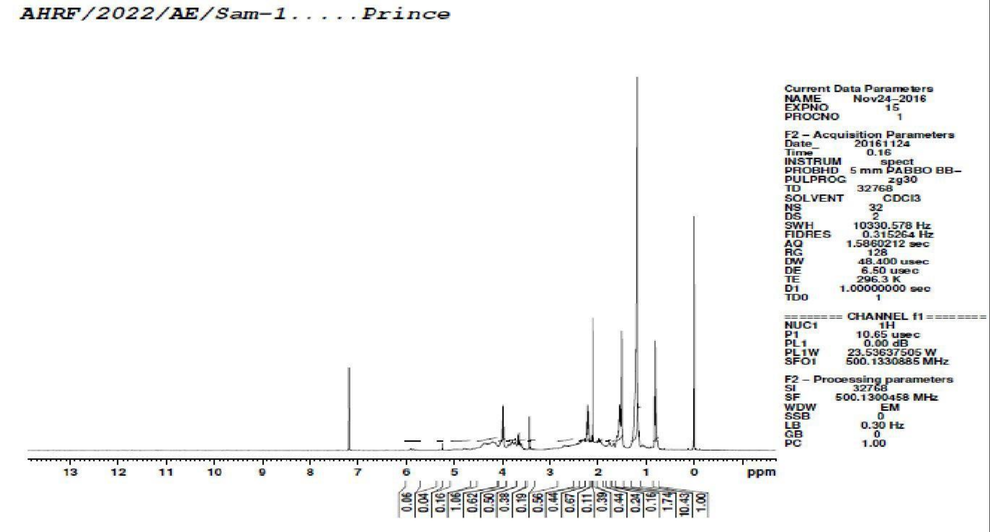

BRUKER NMR 400MHz
^{13}CNMR($CDCl_3$):

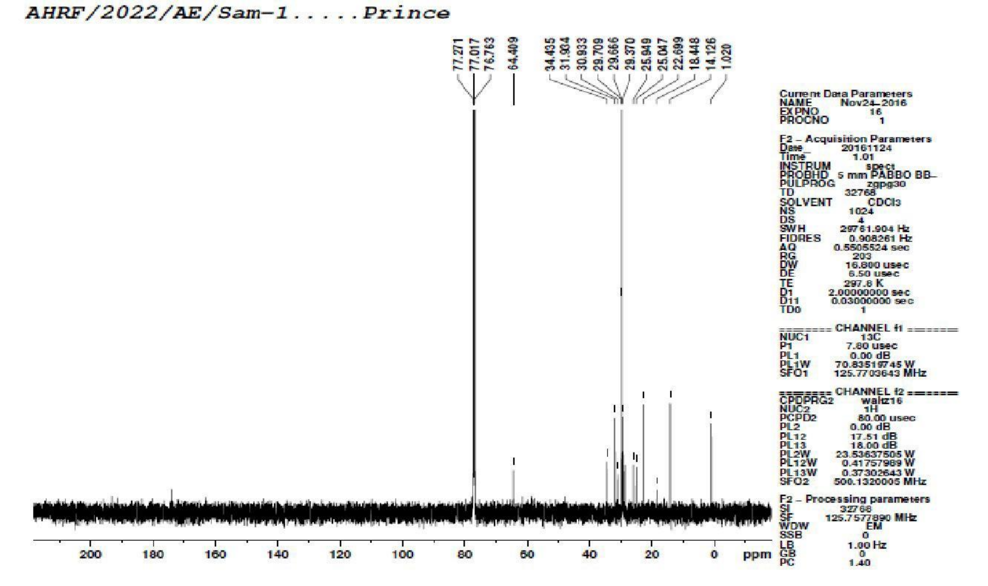

COMPOUND 2 (AE-HX-001-P2)

SPECIFICATON	SPECTRUM
Probable structure	Spinasterol 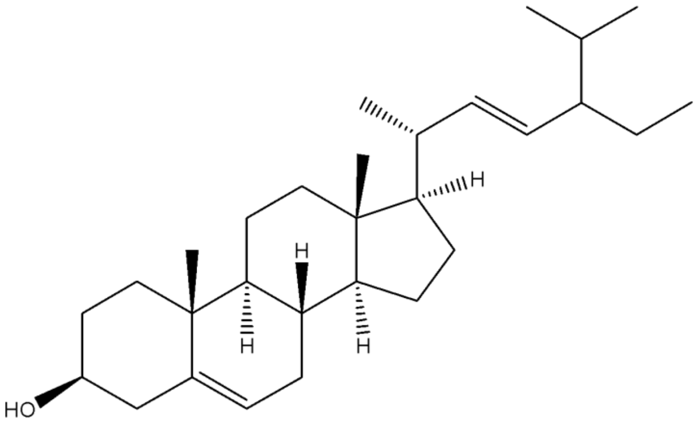
CAMAG HPTLC Scanner III<br>Mobile Phase: Hex: EtoAc (7:3)<br>Rf: 0.74<br>λ max: 203nm	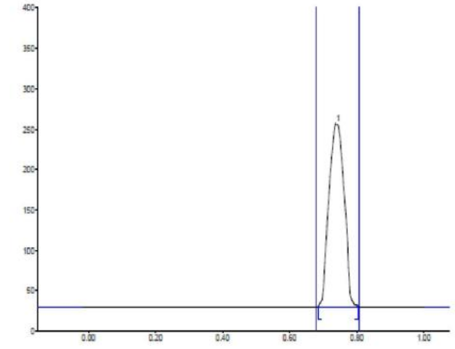 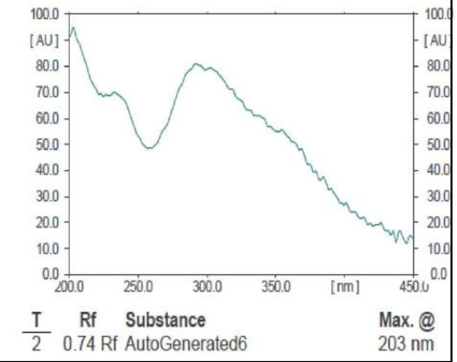
UV spectrum<br>PDA detector<br>λ max: 194nm	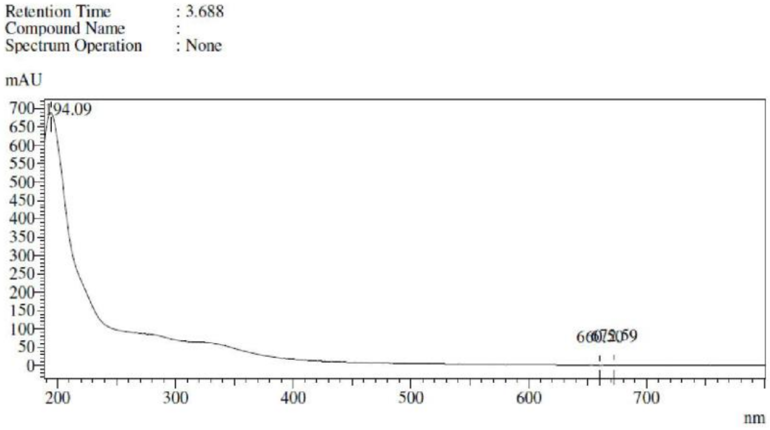

SHIMADZU SPD-20A HPLC<br>Column: Enable C_{18}, 4.6x250mm (5micron)<br>Mobile Phase: ACN:H_2O (15:85) with 0.1% H_3PO_4<br>λ max: 280nm<br>Inj. Vol: 20µl<br>Flow rate: 1.0ml/min<br>Rt: 3.688min	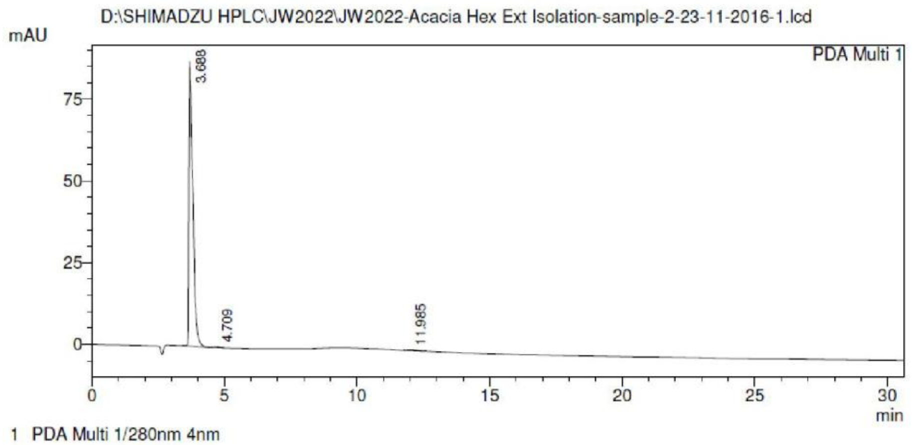
Bruker FT-IR<br>Wavelength scan: 500-4000cm^{-1}	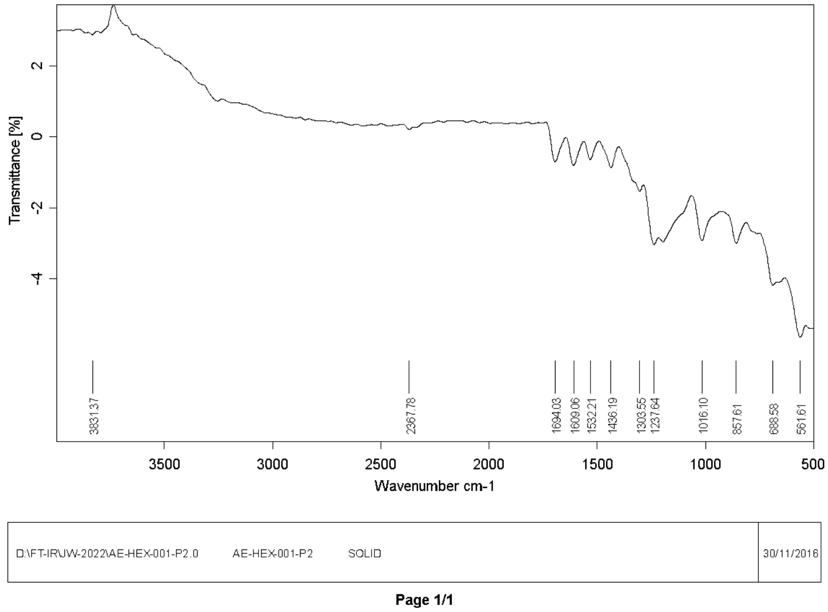
BRUKER NMR 400MHz<br>^{1}HNMR($CDCl_3$):	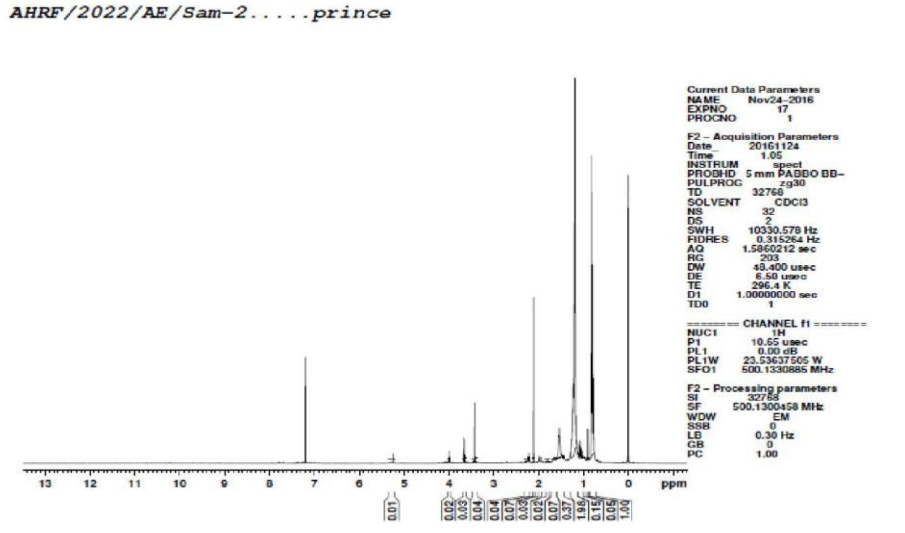

BRUKER NMR 400MHz<br>13CNMR($CDCl_3$):	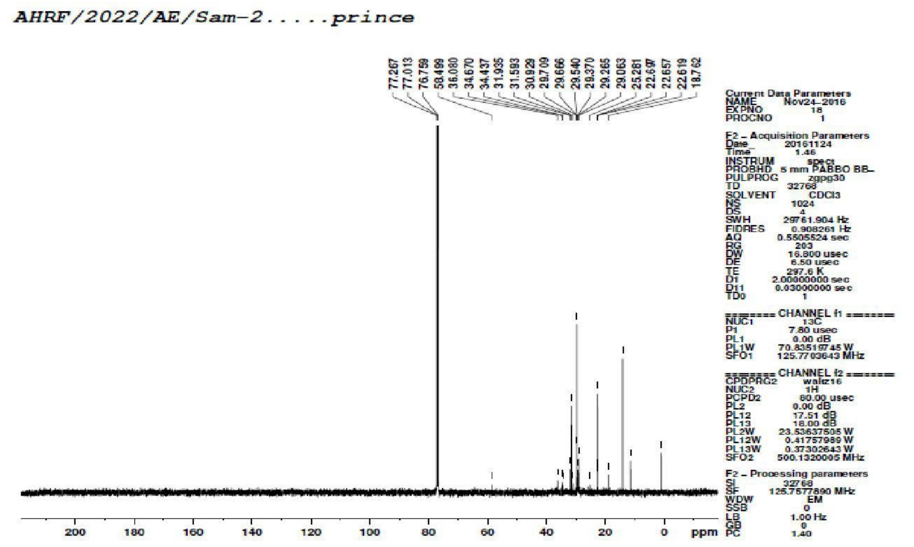

COMPOUND 3 (AE-HX-001-P3)

SPECIFICATON	SPECTRUM
Probable structure	Quinic acid derivative – theogallin 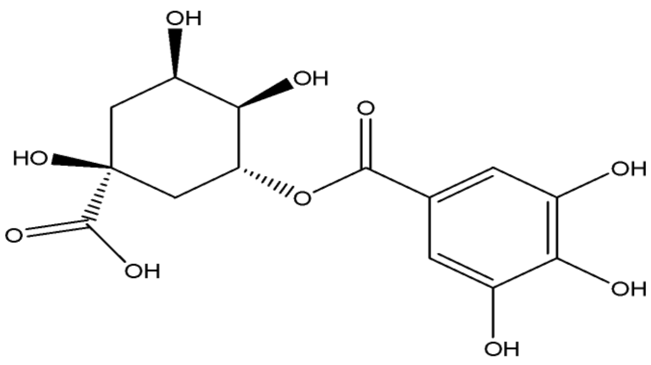
CAMAG HPTLC Scanner III<br>Mobile Phase: Hex: EtoAc (7:3)<br>Rf: 0.61<br>λ max: 202nm	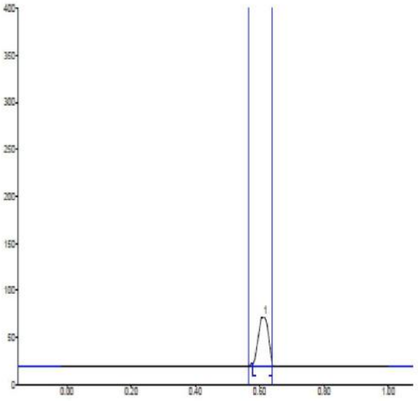 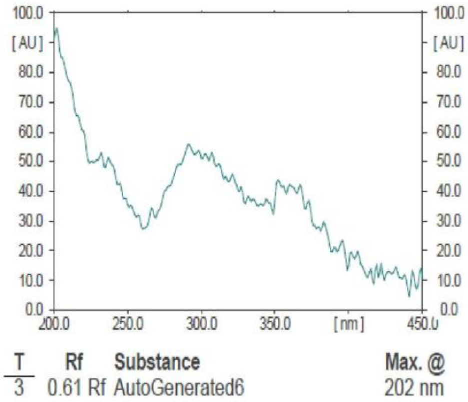

UV spectrum<br>PDA detector<br>λ max: 215nm, 272nm	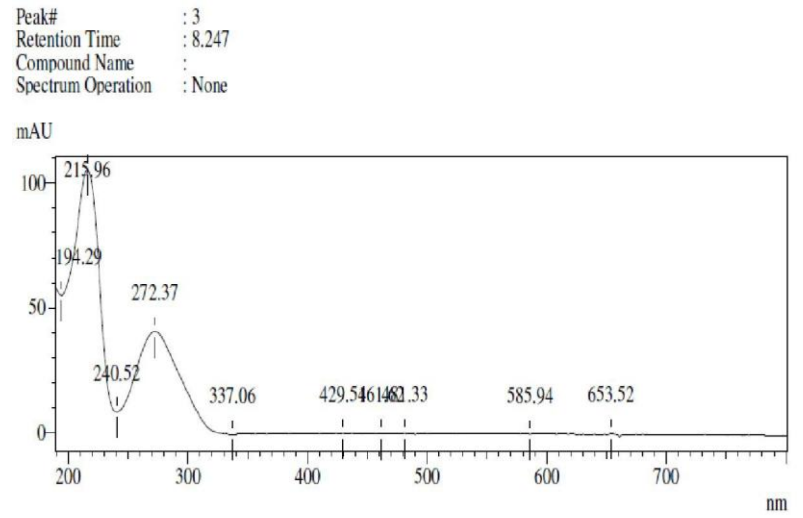
SHIMADZU SPD-20A HPLC<br>Column: Enable C_{18}, 4.6x250mm (5micron)<br>Mobile Phase: ACN:H_2O (<br>λ max: 280nm<br>Inj. Vol: 20µl<br>Flow rate: 1.0ml/min<br>Rt: 8.247min	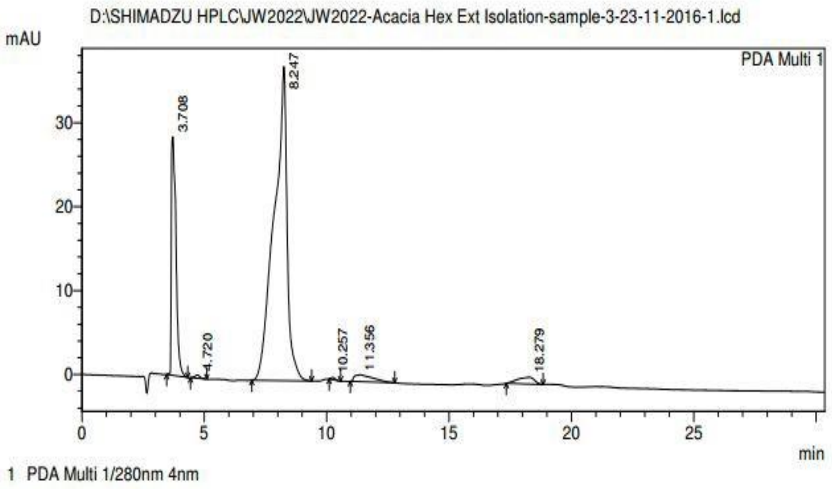
Bruker FT-IR<br>Wavelength scan: 500-4000cm^{-1}	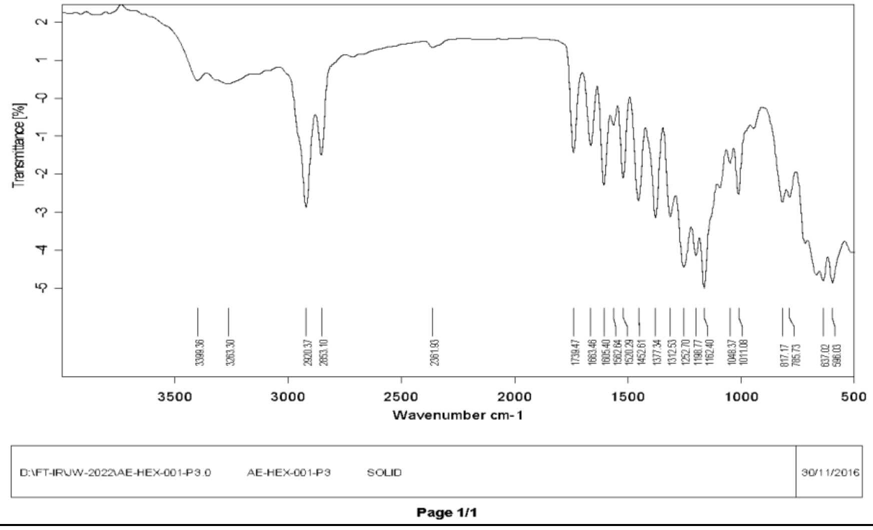

BRUKER NMR 400MHz
$^{1}HNMR(CDCl_3)$:

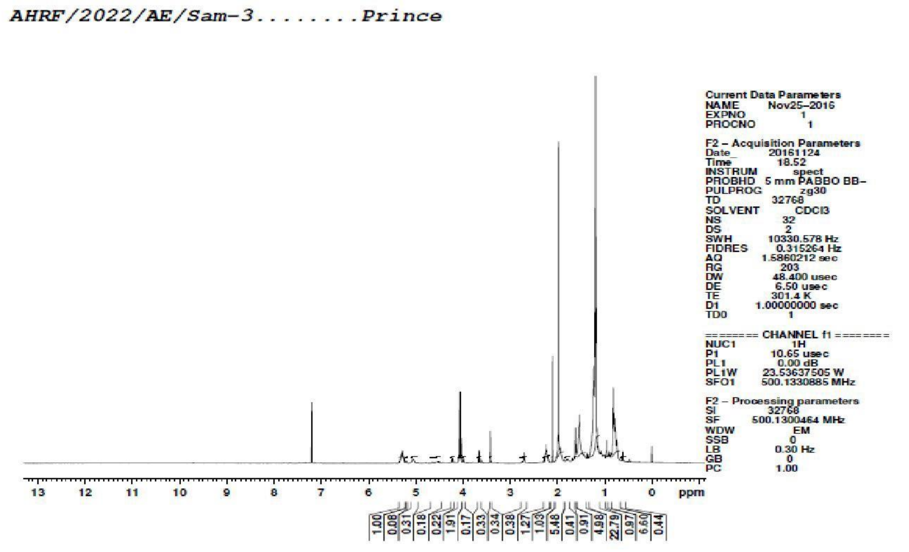

BRUKER NMR 400MHz
$13CNMR(CDCl_3)$:

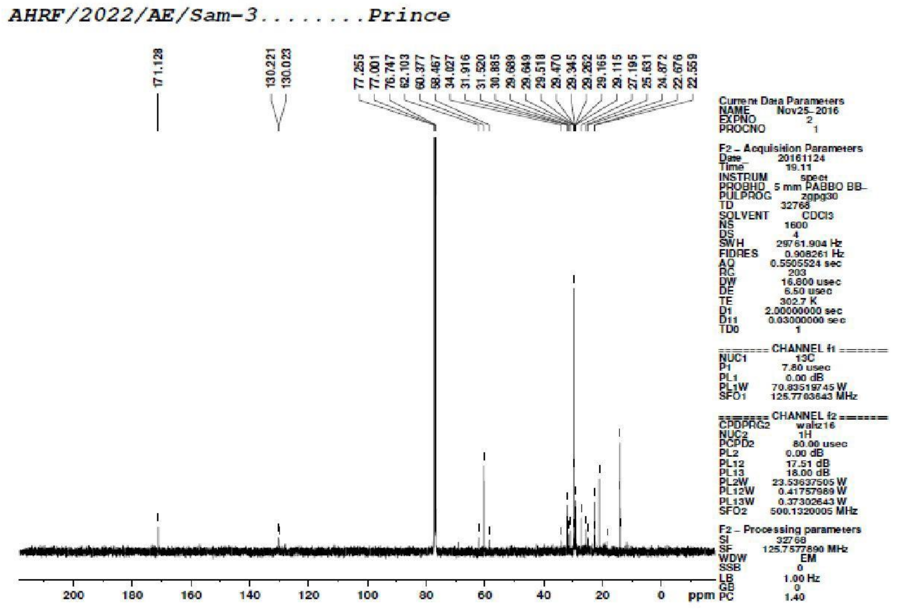

Supplement: Supplementary file 1 [file molecules-27-06400-s001.zip › molecules-1905887-supplementary.pdf]
